# Striatal dopamine D_2/3_ receptors in medication-naïve schizophrenia: an [^123^I] IBZM SPECT study

**DOI:** 10.1017/S0033291720005413

**Published:** 2022-10

**Authors:** Kao Chin Chen, Yen Kuang Yang, Oliver D. Howes, I Hui Lee, Tzung Lieh Yeh, Nan Tsing Chiu, Po See Chen, Anthony S. David, Elvira Bramon

**Affiliations:** 1 Department of Psychiatry, National Cheng Kung University Hospital, College of Medicine, National Cheng Kung University, Tainan, Taiwan; 2 Institute of Behavioral Medicine, College of Medicine, National Cheng Kung University, Tainan, Taiwan; 3 Department of Psychiatry, Tainan Hospital, Ministry of Health and Welfare, Tainan, Taiwan; 4 Department of Psychosis Studies, Institute of Psychiatry, Psychology and Neuroscience, King's College London, London, UK; 5 Institute of Clinical Sciences, Faculty of Medicine, Imperial College London, London, UK; 6 Department of Nuclear Medicine, National Cheng Kung University Hospital, College of Medicine, National Cheng Kung University, Tainan, Taiwan; 7 Department of Psychiatry, National Cheng Kung University, Dou-Liou Branch, Yunlin, Taiwan; 8 Institute of Mental Health, University College London, London, UK; 9 Mental Health Neurosciences Research Department, Division of Psychiatry, University College London, London, UK

**Keywords:** [^123^I] iodobenzamide, dopamine, dopamine receptor, healthy individual, medication-naïve schizophrenia, single-photon emission tomography (SPECT)

## Abstract

**Background:**

The hyper-function of the striatal dopamine system has been suggested to underlie key pathophysiological mechanisms in schizophrenia. Moreover, patients have been observed to present a significant elevation of dopamine receptor availability compared to healthy controls. Although it is difficult to measure dopamine levels directly in humans, neurochemical imaging techniques such as single-photon emission computed tomography (SPECT) provide indirect indices of *in vivo* dopamine synthesis and release, and putative synaptic levels.

**Methods:**

We focused on the role of dopamine postsynaptic regulation using [^123^I] iodobenzamide (IBZM) SPECT. We compared D_2/3_ receptor availability between 53 healthy controls and 21 medication-naive patients with recent-onset schizophrenia.

**Result:**

The mean specific striatal binding showed no significant difference between patients and controls (estimated difference = 0.001; 95% CI −0.11 to 0.11; *F* = 0.00, df = 1, 69; *p* = 0.99). There was a highly significant effect of age whereby IBZM binding declined with advancing age [estimated change per decade of age = −0.01(binding ratio); 95% CI −0.01 to −0.004; *F* = 11.5, df = 1, 69; *p* = 0.001]. No significant correlations were found between the mean specific striatal binding and psychopathological or cognitive rating scores.

**Conclusions:**

Medication-naïve patients with recent-onset schizophrenia have similar D_2/3_ receptor availability to healthy controls. We suggest that, rather than focusing exclusively on postsynaptic receptors, future treatments should target the presynaptic control of dopamine synthesis and release.

## Introduction

Central dopaminergic hyperactivity continues to be one of the key hypotheses for the pathophysiology of schizophrenia (Howes & Kapur, 2009; Howes & Murray, 2014; Seeman, Lee, Chau-Wong, & Wong, 1976). Excess transmission at dopamine receptors and blockade of these receptors to treat psychosis were the primary focus in initial formulations in the 1970s (Matthysse, 1973; Snyder, 1976). Later in the 1990s, a modified dopamine hypothesis of schizophrenia was proposed (Davis, Kahn, Ko, & Davidson, 1991) based on, for example, neuronal lesions in the prefrontal cortex in rats resulting in increased levels of dopamine and in greater dopamine D_2_ receptor density in the striatum (Pycock, Kerwin, & Carter, 1980). The hyper-function of the striatal dopamine system has been suggested to underlie the pathophysiology of the positive symptoms of schizophrenia (Abi-Dargham et al., 2004; Davis et al., 1991; Howes et al., 2009b; McCutcheon, Abi-Dargham, & Howes, 2019; Snyder, 1976). Positive symptoms may be induced by the increased synaptic release of dopamine in the striatum (Breier et al., 1997; Buchsbaum et al., 2006; de Haan et al., 2004; McCutcheon, Beck, Jauhar, & Howes, 2018; Schmitt et al., 2008; Yang et al., 2004). Furthermore, patients also show a significant elevation in striatal synaptic dopamine levels compared to healthy controls (Kegeles et al., 2010; McCutcheon et al., 2018; Slifstein & Abi-Dargham, 2018).

Neurochemical imaging techniques single-photon emission computed tomography (SPECT) and positron emission tomography (PET) provide *in vivo* indices of the different stages of dopamine neurotransmission, including its pre-synaptic synthesis, release into the synapse, and the levels of receptors and transporters (Howes & Kapur, 2009; McCluskey, Plisson, Rabiner, & Howes, 2020).

Elevated dopamine synthesis capacity has been consistently reported in 6-fluoro-(^18^F)-L-3,4-dihydroxyphenylalanine ([^18^F]-DOPA) and L-[b-^11^C]-3,4-dihydroxyphenylalanine ([^11^C]-DOPA) PET studies in schizophrenia, including in first-episode patients (Hietala et al., 1995; Jauhar et al., 2019) and has been shown to predate the onset of schizophrenia in individuals with prodromal psychotic symptoms (Howes et al., 2009b). Synaptic dopamine can be studied using challenge approaches which stimulate its release or deplete synaptic dopamine levels (Egerton, Demjaha, McGuire, Mehta, & Howes, 2010). These approaches are based on the competition between dopamine and radioligands such as raclopride and [^123^I] iodobenzamide (IBZM) for binding to dopamine receptors (Laruelle, 2000), although recent evidence indicates the process is more complex than suggested by a simple competition model (Guo et al., 2010). Studies using challenge approaches have found evidence of increased radiotracer displacement in patients with untreated schizophrenia compared to controls, indicating greater dopamine release (Laruelle, Abi-Dargham, Gil, Kegeles, & Innis, 1999), and increased synaptic dopamine levels (Abi-Dargham et al., 2000; Kegeles et al., 2010; McCutcheon et al., 2018). The dopamine hypothesis of schizophrenia has been revised in light of this neurochemical imaging evidence (Howes & Murray, 2014; McCutcheon et al., 2019).

Indeed, the earlier formulations were built on the findings that antipsychotics work by blocking D_2/3_ receptors, and drugs such as amphetamine which activate the dopamine system, can trigger psychotic symptoms (Abi-Dargham, 2004; Berman, Kuczenski, McCracken, & London, 2009; Curran, Byrappa, & McBride, 2004; Howes et al., 2009a). Based on early findings of an elevation in striatal D_2/3_ receptor availability in schizophrenia (Wong et al., 1986), there was an initial focus on the D_2_ receptor. However, subsequent studies of D_2/3_ receptor availability in schizophrenia have been inconsistent, whilst there is no difference *v.* controls in dopamine transporter availability (Chen et al., 2013; Howes et al., 2012). One factor in many of the studies that could explain the inconsistent findings is prior antipsychotic treatment, which may upregulate D_2/3_ receptor levels and increase variability in patients (Brugger et al., 2020). There is therefore a need for large studies of medication-naïve first-episode patients to determine whether dopamine receptor availability abnormalities are associated with the onset of the illness.

Cognitive impairments have consistently been found in patients with schizophrenia compared to healthy individuals (Elvevag, Weinberger, Suter, & Goldberg, 2000; Lencz et al., 2014; Ranlund et al., 2018) and may be considered a core aspect of the clinical syndrome. There is growing evidence that cognition is a pathway through which genetic variation influences schizophrenia risk (Calafato & Bramon, 2019; Toulopoulou et al., 2019). Indeed, a genome-wide association meta-analysis of human cognition including over 129 000 participants showed that intelligence has a strong protective effect on schizophrenia risk (Savage et al., 2018). The Wisconsin Card Sorting Test (WCST) and Continuous Performance Task (CPT) are commonly used to evaluate the aspects of patients' cognitive functions (Bellani & Brambilla, 2008; Elvevag et al., 2000; Everett, Lavoie, Gagnon, & Gosselin, 2001; Green, Satz, Ganzell, & Vaclav, 1992). The WCST was found to relate to genetic variation in dopamine receptors (Rybakowski et al., 2005, 2006), and CPT was influenced by the estimates of dopamine release in patients with schizophrenia (Braver, Barch, & Cohen, 1999; Cohen & Servan-Schreiber, 1993). Medication-free patients with schizophrenia show reduced prefrontal cortical dopamine release while performing cognitive tasks such as WCST, which supports the frontal hypo-dopaminergic hypothesis of cognitive symptoms in schizophrenia, and suggests a differential regulation of striatal dopamine release in associative regions (Rao et al., 2019; Slifstein et al., 2015). We hypothesize that patients with higher striatal D_2/3_ receptor availability, which indicates higher dopamine release, should have better cognitive performance (Fagerlund et al., 2013).

Our study focused on post-synaptic dopamine regulation using [^123^I] IBZM SPECT. We set out to compare D_2/3_ post-synaptic receptor availability between 53 healthy controls and 21 drug-naïve patients with recent-onset schizophrenia. The relationships between D_2/3_ receptor availability and both cognitive function and clinical symptoms were also investigated.

## Methods

### Sample

All study participants were living in Tainan City, the fifth largest in Taiwan with a population of 1 880 906 (Bureau of civil affairs, Tainan city government, 2019). A total of 21 medication-naïve first-episode patients with schizophrenia were recruited from the psychiatric outpatient clinic of the National Cheng Kung University Hospital. This included 11 patients and one control from our previous study (Yang et al., 2004). Fifty-three healthy community residents of Tainan City were recruited as volunteers through research advertisements. All participants were right-handed. Patients were recruited from August 2001 to April 2005 and controls were recruited from June 1999 to April 2005. All participants including controls were interviewed by senior psychiatrists who have been practicing for more than 10 years, using the Chinese version of the Mini International Neuropsychiatric Interview (Sheehan et al., 1998), to ensure that the controls were free of any Axis I or Axis II psychiatric disorders and to confirm the diagnosis for patients. The Positive and Negative Syndrome Scale (PANSS) of patients with schizophrenia was rated by one psychiatrist. Brain magnetic resonance images (MRI) and blood biochemical profiles of all controls were assessed and showed no abnormalities. The mean duration of illness was 10.7 months (s.d. = 20.7, median = 2.0, interquartile range = 11.8 months).

Before any procedure was performed, written informed consent was obtained from each of the participants after a complete explanation of the study. The Ethical Committee for Human Research at the National Cheng Kung University Hospital approved the study. Inclusion criteria: (i) patients should fulfill DSM-IV criteria for schizophrenia; (ii) age between 17 and 60; (iii) no physical illness and with stable vital signs; (iv) participants never received any antipsychotic or antidepressant treatment and were free of any psychotropic medication at the time of testing. Exclusion criteria for all participants: (i) other co-morbid psychiatric illnesses, or neurological illnesses; (ii) evidence of substance abuse/dependence as assessed during the clinical interview with the research psychiatrist, at time of enrollment; (iii) intellectual disability; (iv) all female participants of child-bearing age had to take an acceptable form of contraceptive throughout the study, in order to be included and underwent an instant urine pregnancy test prior to starting the experiments; (iv) all patients who were deemed at risk of acute suicide/self-harm were excluded.

### Assessment battery

Before receiving any treatment, patients underwent the baseline assessments within 7 days of entering the study including SPECT, psychopathology scales and cognitive testing. Healthy controls received the same assessments. Baseline assessments are described below.

### Measurement of striatal dopamine D_2/3_ receptor density

Before SPECT examination with [^123^I] IBZM, the thyroid gland was protected with 9 ml of Lugol's solution. For brain SPECT imaging, each subject was intravenously administered 185 MBq (injected mass of IBZM: 8.2 ng; specific radioactivity 8900 MBq/nmol) of [^123^I] IBZM (Institute of Nuclear Energy Research, Lungtan, Taiwan) in a quiet environment approximately 10 min after setting the intravenous lines. The imaging was initiated approximately 120 min later, and 30 min of imaging data were collected during the procedure. To avoid tilt and misalignment, we carefully positioned participants and monitored them during scanning, and used a head holder to further reduce movement artifacts. Participants were informed of the necessity to avoid head movement. Sinograms were reviewed blind to diagnosis to determine whether post-acquisition correction for head movements was needed. Movement correction was conducted using the motion correction software ICON (Siemens, version 8.5 KB21).

**Fig. 1.**
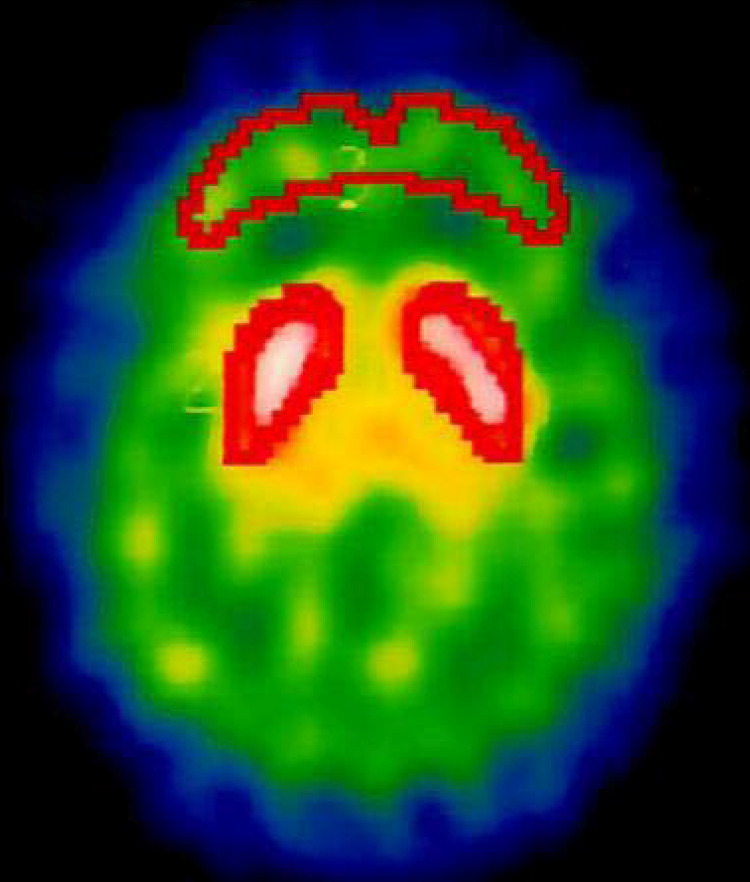
The regions of interest (ROIs) were placed over the striatum and the frontal cortex. The ROIs were drawn directly on the SPECT images by an experienced nuclear-medicine physician who was blind to the participants' clinical status and data.

We used a triple-headed rotating *γ* camera (Multispect 3; Siemens, Hoffman Estates, IL, USA) with ultra-high-resolution fan-beam collimators, which yields an image resolution of approximately 8.5 mm for the full width half maximum (FWHM). The SPECT images were acquired over a circular 360° rotation, with 120 steps, at a rate of 50 s per step, in a 128 × 128 × 16 matrix. The images were then reconstructed using Butterworth and Ramp filters (Friston et al., 1990) (cut-off frequency = 0.3 Nyquist, power factor = 7), with attenuation according to Chang's method (Chang, 1978). The reconstructed transverse images were realigned parallel to the canthomeatal line; slice thickness = 2.89 mm. For semi-quantitative analyses, six consecutive transverse slices on which the striatum was best visualized were combined to obtain a 17.34 mm-thick slice. Then regions of interest (ROIs) were placed over the striatum and the frontal cortex (see Fig. 1). The ROIs were drawn directly on the SPECT images by an experienced nuclear-medicine physician who was blind to the participants' clinical status and data. During this process, the participants' MRIs (GE, SIGNA CV-I, 1.5T, WI, USA), obtained within 2 weeks after SPECT examination, were used as a visual reference to determine the areas of the ROI. The sizes of all ROI were at least twice those of the FWHM. The specific striatal [^123^I] IBZM binding was calculated as the mean count in the striatal ROI minus the mean count in the frontal region divided by the mean count in the frontal region [(St−F)/F] and this ratio represents striatal D_2/3_ availability (Brucke et al., 1991; Toyama, Ichise, Ballinger, Fornazzari, & Kirsh, 1993).

### Psychopathology ratings

On the day of recruitment, we collected standardized psychopathology ratings using the Global Assessment of Functioning (GAF), which ranges 0–100 from poorest to optimal functioning (Hopper & Wanderling, 2000) and the PANSS, with 30 psychotic symptom and general psychopathology items (range 30–210) from least to most severely symptomatic (Kay, Fiszbein, & Opler, 1987).

### Cognitive function assessments

Patients' executive function and attention/vigilance were assessed using the Wisconsin Card Sorting Test (WCST) and the Continuous Performance Test (CPT), respectively.

#### Wisconsin Card-Sorting Test (WCST)

A computerized version of the WCST was administered by an experienced clinical neuropsychologist. Definitions of indices were as described in the WCST manual (Heaton, Chelune, Talley, Kay, & Curtiss, 1993). The number of categories completed and perseverative errors were used to assess the performance (Stratta et al., 1997; Volkow et al., 1998).

#### Continuous Performance Test (CPT)

The CPT is a psychological test that primarily measures attention (Chen, Hsiao, Hsiao, & Hwu, 1998; Hsieh et al., 2005). In this version, the critical stimulus was a particular sequence of two stimuli out of the available set (AX task: subjects were asked to respond whenever the number ‘9’ was preceded by the number ‘1’). Each test session began with a 2 min practice. During the test, numbers from 0 to 9 were randomly presented for 50 ms each, at a rate of one per second. A total of 331 trials, 34 (10%) of which were target stimuli, were presented over 5 min. Subject responses were recorded automatically (Sunrise Systems, version 2.20, Pembroke, MA, USA) (Smid, de Witte, Homminga, & van den Bosch, 2006).

### Statistical analyses

The main aim of our study was to assess the differences between patients and controls in the specific striatal [^123^I] IBZM binding, considering both the left and right striatum measures. Linear mixed modeling was used to compare [^123^I] IBZM binding between the two participant groups. To allow for possible group differences between left and right sites, we tested a group by laterality interaction. On the basis of previous literature, we considered that age, sex, and tobacco smoking are potential confounders and therefore included these as covariates in the analysis (Chen et al., 2005; Kuikka, Tiihonen, Karhu, Bergstrom, & Rasanen, 1997; Volkow et al., 1998; Yang et al., 2008). The models included subject-varying intercepts to acknowledge the correlation between the two repeated measures per participant. We expanded the above model to test the interaction of group by age or group by sex, respectively.

Our subsidiary aim was to explore the association between the specific striatal [^123^I] IBZM binding and psychopathology ratings or cognitive performance in patients using Spearman's *ρ* correlations.

Demographic differences between patients and controls were examined with χ^2^ tests for categorical variables or with Student's *t* tests for continuous variables. For the latter, Levene's test was used to assess the assumption of the equality of variances. Diagnostic plots as well as one-sample Kolmogorov–Smirnov tests were used to test for normality.

We report uncorrected *p* values for all analyses. However, since we examined the influence of striatal [^123^I] IBZM binding on 10 key parameters (clinical group, four PANSS scores, four cognitive measures, and GAF), we adjusted for multiple testing and our statistical significance was established at *p* ＜ 0.005 (statistical trend *p* < 0.01). We used SPSS version 20.0 for Windows (SPSS Inc., Chicago, IL, USA) for all analyses.

## Results

The demographic characteristics of the patient and control groups are summarized in Table 1. Patients and controls had a similar sex distribution and tobacco smoking habits. However, compared to the controls, the patients were significantly younger (*t* = −2.33, df = 72; *p* = 0.02), less likely to be married (χ^2^ = 7.71; *p* = 0.005), and had fewer years of education (*t* = −3.44, df = 71; *p* = 0.001). The ratio of specific striatal binding in both patients and controls was normally distributed (Kolmogorov–Smirnov test was not significant and diagnostic plots showed no departure from normality).

**Table 1.** Demographic characteristics of participants

	Schizophrenia (*n* = 21)	Control (*n* = 53)	Statistical test			
	Mean (s.d.)	Mean (s.d.)	*t*/*x^2^*	df	*p*	Cohen's *d*/*φ* coefficient
Age, years (s.d.) range	26.7 (9.0) 17–46	32.8 (10.6) 20–54	−2.33	72	0.02*	0.599
Sex (male/female)	11/10	28/25	0.001	1	0.97	0.004
Smoking status (yes/no)	5/16	10/43	0.23	1	0.63	0.056
Marital status (M/S)	2/19	23/30	7.71	1	0.005**	0.323
Years of education range	12.8 (2.0) 9–16	14.7 (2.2) 9–19	−3.44	71	0.001**	0.885

M, married or living with a partner; S, single, divorced or separated.

*p < 0.05; **p < 0.01.

**Fig. 2.**
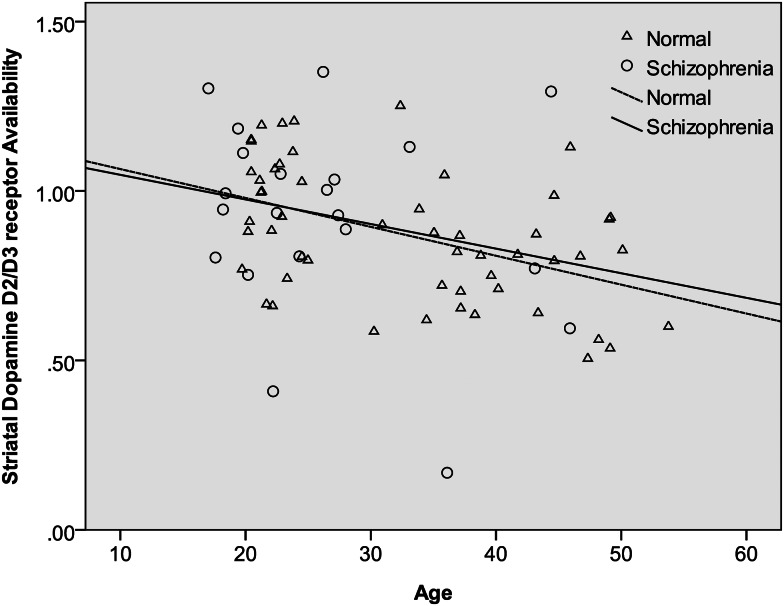
The relation between age and striatal dopamine D_2/3_ receptor availability [(St−F)/F] in patients with schizophrenia and controls. As described in the main analyses, having adjusted for the main effects of group, sex, and tobacco smoking, there was a significant decline of IBZM binding with advancing age but no significant interaction between the age and group. This graph shows regression lines describing the relationship between [^123^I] iodobenzamide binding and age within patients and controls separately. The almost parallel lines illustrate the similar rates of decline in the two groups.

After controlling for age, sex, and tobacco smoking, the mean specific striatal binding showed no significant difference between patients and controls (estimated difference = 0.001; 95% CI −0.11 to 0.11; *F* = 0.00, df = 1, 69; *p* = 0.99). These results are summarized in Table 2. In the same, there was a significant main effect of laterality, whereby the right side had a higher ratio of the specific striatal binding than the left side (estimated difference = 0.03; 95% CI 0.007–0.048; *F* = 7.28, df = 1, 73; *p* = 0.009). The group by laterality interaction was not significant and was therefore dropped from our model (*F* = 0.01, df = 1, 72; *p* = 0.91). There was no significant effect of sex (estimated difference = −0.01; 95% CI −0.12 to 0.10; *F* = 0.03, df = 1, 69; *p* = 0.86). Similarly, tobacco smoking did not have a significant influence on IBZM binding (estimated difference = 0.06; 95% CI −0.08 to 0.19; *F* = 0.65, df = 1, 69; *p* = 0.42). Finally, there was a highly significant effect of age whereby IBZM binding declined with advancing age (estimated binding ratio change per decade of age = −0.01; 95% CI −0.01 to −0.004; *F* = 11.5, df = 1, 69; *p* = 0.001). Of note there was no significant interaction between age and group (*F* = 0.02, df = 1, 68; *p* = 0.89) or between group and sex (*F* = 0.17, df = 1, 68; *p* = 0.68), indicating that the age decline in IBZM was similar in patients and controls as well as in both sexes. These findings are summarized in Table 2 and Fig. 2.

**Table 2.** Dopamine D_2/3_ receptor availability [(St−F)/F] by group and sex

		Total			Right			Left		
	*N*	Mean	(s.d.)	Range	Mean	s.d.	Range	Mean	s.d.	Range
Group										
Schizophrenia	21	0.93	(0.29)	0.17–1.35	0.94	(0.28)	0.20–1.38	0.91	(0.31)	0.14–1.43
Control	53	0.87	(0.19)	0.51–1.25	0.88	(0.19)	0.56–1.27	0.86	(0.21)	0.45–1.24
95% CI^a^		−0.11, 0.11			−0.11, 0.11			−0.13, 0.12		
Sex										
Males	39	0.87	(0.25)	0.17–1.30	0.89	(0.24)	0.20–1.38	0.85	(0.26)	0.14–1.36
Females	35	0.91	(0.20)	0.41–1.35	0.91	(0.18)	0.43–1.27	0.90	(0.21)	0.39–1.43
95% CI^a^		−0.12, 0.10			−0.08, 0.19			−0.14, 0.10		

[(St−F)/F]: mean count in the striatal region minus the mean count in the frontal region divided by the mean count in the frontal region.

a Independent variables included group, age, sex, and tobacco smoking.

Among the 21 patients included, 19 patients completed the GAF, and 19 patients completed the PANSS scales. No significant correlations were found between the mean specific striatal binding and psychopathological rating scores. A total of 17 patients completed WCST and 16 patients completed CPT. There was suggestive evidence for a positive correlation between the mean specific striatal binding and the scores of the number of categories completed in WCST (*ρ* = 0.54, *p* = 0.02) (Table 3), however this did not survive correction for multiple testing.

**Table 3.** Spearman's *ρ* correlations of D_2/3_ receptor availability [(St−F)/F] and psychopathology in the schizophrenia group

		PANSS				GAF	WCST		CPT	
		Positive	Negative	General psychopathology	Total		Perseverative errors	Number of categories completed	Unmask *d*'	Mask *d*'
*N*		19	19	19	19	19	17	17	18	16
Mean (s.d.)		18.9 (5.8)	17.5 (5.1)	28.5 (5.9)	64.8 (11.3)	48.0 (14.0)	11.1 (5.2)	2.4 (1.1)	3.6 (1.1)	3.1 (1.1)
Range		7–29	11–29	19–40	43–86	28–75	4–22	1–4	1.7–4.9	1.2–4.9
IBZM binding ratio	*ρ*	−0.11	0.41	0.18	0.24	0.37	−0.29	0.54	0.11	0.43
	*p*	0.65	0.08	0.45	0.32	0.12	0.26	0.02	0.67	0.10

IBZM, [^123^I] iodobenzamide; PANSS, Positive and Negative Syndrome Scale; GAF, Global Assessment of Functioning; WCST, Wisconsin Card Sorting Test; CPT, Continuous Performance Task.

[(St−F)/F]: mean count in the striatal region minus the mean count in the frontal region divided by the mean count in the frontal region.

*p < 0.005, adjusted for multiple testing.

We used the median, 0.95, of IBZM binding ratio [(St−F)/F, D_2/3_ receptor availability] as the cut-off point to divide patients into low and high dopamine binding groups with no differences in age, sex, tobacco smoking (high: *n* = 10, low: *n* = 11) (Tanaka, 2006) based on the concept of hyperdopaminergic/normo-dopaminergic subtyping in schizophrenia (Howes & Kapur, 2014). We found no evidence of group differences in the masked *d*’ between the high dopamine binding group (*n* = 9) and the low dopamine binding group (*n* = 7) (Mann–Whitney *U* = −1.74; *p* = 0.08). Similarly, no significant difference was found in WCST performance between the two groups.

There were no significant differences between patients recruited in 2004 (*n* = 11) and after 2004 (*n* = 10) in demographic or imaging measures or PANSS scores (positive, negative, general psychopathology, sum, *p* = 0.08, 0.48, 0.02, 0.06). We found weak evidence for better WCST scores (perseveration errors and number of categories completed, *p* = 0.07 and 0.06), and CPT unmask *d*’ score (*p* = 0.07) in patients recruited after 2004 (online Supplementary Table S1).

In conclusion, these findings provide some support for our hypothesis that, amongst people with schizophrenia, a higher dopamine release is associated with better cognitive performance (Fagerlund et al., 2013).

## Discussion

Our study shows that the specific striatal binding ratio of medication-naive patients with schizophrenia was not significantly different from that of healthy controls, and we found no evidence of changes in D_2/3_ receptor availability. Our findings are consistent with previous studies conducted at baseline before treatment. Of note, not all the patients in these preceding experiments were medication-naïve, but those patients recruited in the study of Wulff et al. were completely medication-naïve (Abi-Dargham et al., 2000; Corripio et al., 2011; Wulff et al., 2015).

Previous literature on D_2_ receptor availability showed diverse findings. Indeed, a meta-analysis found a small (Cohen's *d* = 0.26) yet significant elevation of D_2/3_ receptors in schizophrenia. It also showed D_2/3_ receptor upregulation is not detected in antipsychotic-naïve patients (Howes et al., 2012), which is fully consistent with our data. Other studies reported no evidence of major alterations in dopamine D_2/3_ receptors in patients with schizophrenia (Kegeles et al., 2010; Slifstein & Abi-Dargham, 2018). Thus, while previous imaging studies were inconsistent with regards to D_2/3_ dysfunction and varied depending on clinical characteristics and imaging methods, in light of our findings in medication-naïve patients, these D_2/3_ availability abnormalities are likely to be confounded by antipsychotic medication.

Our research has shown that aging has a powerful influence on both pre- and post-synaptic dopaminergic function. In a previous study, we showed that the specific uptake of [^99m^Tc]-TRODAT-1, a radiotracer for the dopamine transporter, decreases with advancing age, and that this aging decline was observed both in controls and patients, but was faster amongst the antipsychotic-naïve patients with first-episode schizophrenia (Chen et al., 2013). Furthermore, the density of striatal dopamine D_2/3_ receptors also declines with age in healthy individuals (Chen et al., 2005). Finally, in this current study, our methodology was sensitive enough to identify a highly significant effect of age whereby IBZM binding declines with advancing age and it does so at a similar rate in both patients with schizophrenia and controls.

We found suggestive evidence that those patients with higher dopamine release may have better cognition. Our findings support the concept of hyperdopaminergic/normo-dopaminergic subtyping in schizophrenia (Howes & Kapur, 2014) that proposed there are differences in the dopamine system between patients who respond to antipsychotic drugs and those who do not. Although there is a consistent alteration in dopaminergic function in schizophrenia, evidence of heterogeneity is also reported (Howes et al., 2012), higher baseline dopamine metabolite levels are generally associated with good subsequent response to antipsychotic treatment, whereas lower dopamine metabolite levels are associated with poor response (Yoshimura, Ueda, Shinkai, & Nakamura, 2003). Those medication-naïve hyperdopaminergic patients showed tentative evidence of better CPT performance, which suggests cognitive differences could exist in medication-naïve patients with different D_2/3_ receptor availability beside the severity of symptoms. There was also some evidence that a better performance in WCST correlated with a higher mean specific striatal binding (dopamine D_2/3_ receptor availability) in our study, thus also supports the aforementioned findings. Our results did not show significant correlations between the mean specific striatal binding (dopamine D_2/3_ receptor availability) and psychopathological rating scores, which is consistent with our previous study of striatal dopamine transporter availability (Chen et al., 2013). We hypothesize that could be influenced by the complex dysfunction of a specific dorsal fronto-striato-thalamic circuit, linking different brain areas across different stages of psychosis, so simultaneously also affecting cognitive functions (Dandash, Pantelis, & Fornito, 2017; Fagerlund et al., 2013; Okubo et al., 1997). Increased striatal dopamine signaling and impaired integration of cortical inputs into the striatum could affect cognitive components involved in the behaviors of patients with schizophrenia (Conn, Burne, & Kesby, 2020), but may not completely reflect the whole clinical symptoms. Our results support the hypothesis that dysfunction of cortico-striato-thalamic circuits influences the pathogenesis of psychosis, which may also implicate global cognitive deficits in schizophrenia (Dandash et al., 2017).

A strength of our study is that we recruited a relatively large and clinically homogenous sample, where all the patients were medication-naive and at a similar early stage of their illness. Our controls recruited from the community had a similar gender distribution, but were significantly older, and had spent significantly more time in education. These differences are similar to the previous studies of patients with schizophrenia and other psychotic disorders compared to healthy controls (Loughland et al., 2010).

Decreases in either brain volume or dopamine transporter availability in the striatum have been reported in tobacco smokers (Brody et al., 2004; Yang et al., 2008). Our patient and control groups were not significantly different in smoking status, and our previous study has shown no significant difference in striatal dopamine receptor availability between smokers and controls (Yang et al., 2006); hence, smoking is unlikely to have confounded our results. Nevertheless, all our analyses were adjusted for tobacco smoking in this study.

One potential limitation of our study is that the ROIs were manually drawn directly on the SPECT image. While there is the potential for observer bias using this approach, we avoided this by ensuring that the ROIs were delineated independently of the clinical assessment and blind to the subject's clinical group. We delineated the ROI on the SPECT image rather than on the MRI because this does not require the ROI to be transformed from MRI to SPECT space. However, studies have found similar results using both approaches (Inoue et al., 2004; Wang et al., 1996). Furthermore, we used the frontal cortex as the reference region, since no direct evidence indicates cortical dopaminergic alterations in patients with schizophrenia (Kambeitz, Abi-Dargham, Kapur, & Howes, 2014). Another potential limitation is that patients recruited in our study for SPECT imaging had to be relatively cooperative in order to complete the procedure. This is an issue for most imaging studies and may potentially affect the generalizability of our findings to those with more florid disorder. We did not differentiate the subtypes of patients with schizophrenia in our study, which could limit information for further evaluation between specific clinical symptomatology and striatal dopamine D_2/3_ receptor availability (Hietala et al., 1999; McCutcheon, Krystal, & Howes, 2020).

Intriguingly, the evidence from molecular genetics continues to implicate post-synaptic D_2_ receptors in schizophrenia. A recent meta-analysis found that genetic variants coding for D_2_ receptors (C957T polymorphism) constitute a risk factor for schizophrenia especially in Caucasian populations (Liu et al., 2014). Similarly, a landmark genome-wide association study identifying over 100 loci associated with schizophrenia found significant hits implicating the dopamine D_2_ receptor gene indicating its possible role in the etiology of the disease (Flint & Munafo, 2014; Ripke et al., 2014; Ripke, Walters, & O'Donovan, 2020).

Future research will need to characterize the mechanisms through which genetic variants in dopamine receptors influence schizophrenia susceptibility and imaging biomarkers obtained through PET or SPECT, especially when used to study unaffected relatives and populations with increased risk for the disease. Finally, as suggested by Howes et al. (2012) and consistent with this study, future schizophrenia treatments should target the presynaptic control of dopamine synthesis and release rather than focus exclusively on post-synaptic receptors, and further focus on other striatal neurochemistry such as non-dopaminergic neurotransmitter systems that may contribute to dopaminergic dysfunction (McCutcheon et al., 2019).

In conclusion, our imaging evidence does not support a major dopaminergic abnormality in schizophrenia affecting post-synaptic dopamine receptors, although in this study, we did not investigate pre-synaptic synthesis capacity and release (Abi-Dargham, van de Giessen, Slifstein, Kegeles, & Laruelle, 2009; Howes et al., 2012). Both the previous literature and these findings suggest that any enhanced post-synaptic D_2/3_ receptor availability is likely to be secondary to antipsychotic treatment rather than the illness itself.

## Supplementary material

For supplementary material accompanying this paper visit https://doi.org/10.1017/S0033291720005413. click here to view supplementary material
