# Supplementary material for: Striatal dopamine D2/3 receptors in medication-naïve schizophrenia: an [123I] IBZM SPECT study
Source: Psychol Med. 2021 Mar 8;52(14):3251–9. doi: 10.1017/S0033291720005413 (PMC9693693; doi:10.1017/S0033291720005413)
Supplement: Supplementary file 1 [file S0033291720005413sup001.docx]

**Supplementary table 1.** The comparison between patients with schizophrenia recruited in 2004 and after 2004.

|  | After 2004 | | | In 2004 | | | *P of Mann-*  *Whitney U* | Cohen's d |
| --- | --- | --- | --- | --- | --- | --- | --- | --- |
|  | N | Mean | SD | N | Mean | SD |  |  |
| Age | 10 | 28.16 | 7.62 | 11 | 25.33 | 10.19 | 0.18 | 0.31 |
| Years of education | 9 | 13.33 | 2.50 | 11 | 12.27 | 1.42 | 0.32 | 0.54 |
| PANSS Positive | 8 | 21.75 | 4.27 | 11 | 16.82 | 6.08 | 0.08 | 0.91 |
| PANSS Negative | 8 | 16.88 | 5.74 | 11 | 17.91 | 4.74 | 0.48 | 0.20 |
| PANSS General psychopathology | 8 | 32.50 | 5.55 | 11 | 25.55 | 4.27 | 0.02 | 1.44 |
| PANSS Sum | 8 | 71.13 | 9.34 | 11 | 60.27 | 10.62 | 0.06 | 1.07 |
| GAF | 8 | 48.75 | 11.47 | 11 | 47.36 | 16.03 | 0.93 | 0.10 |
| WCST Perseveration Errors | 8 | 9.13 | 5.84 | 9 | 12.78 | 4.12 | 0.07 | 0.73 |
| WCST Number of Categories Completed | 8 | 2.88 | 0.99 | 9 | 1.89 | 1.05 | 0.06 | 0.97 |
| CPT unmask d' | 9 | 4.05 | 1.10 | 9 | 3.18 | 0.98 | 0.07 | 0.84 |
| CPT mask d' | 8 | 3.07 | 1.22 | 8 | 3.14 | 0.99 | 0.92 | 0.06 |
| Dopamine D_2/3_ receptor availability [(St-F)/F] |  |  |  |  |  |  |  |  |
| Total | 10 | 0.94 | 0.37 | 11 | 0.91 | 0.20 | 0.32 | 0.10 |
| Right | 10 | 0.94 | 0.35 | 11 | 0.94 | 0.21 | 0.40 | 0.00 |
| Left | 10 | 0.95 | 0.41 | 11 | 0.88 | 0.21 | 0.29 | 0.22 |
